# Hospital caseload thresholds for improved perioperative outcomes during transurethral resection or enucleation of the prostate: results from the GRAND study

**DOI:** 10.1007/s00345-026-06559-y

**Published:** 2026-06-25

**Authors:** Nikolaos Pyrgidis, Gerald Bastian Schulz, Philipp Weinhold, Yannic Volz, Michael Atzler, Leo Federico Stadelmeier, Iason Papadopoulos, Christian Stief, Julian Marcon, Patrick Keller

**Affiliations:** Department of Urology, University Hospital, LMU Munich, Marchioninistraße 15, Munich, 81377 Germany

**Keywords:** Benign prostatic hyperplasia, Laser enucleation, Transurethral resection of the prostate, Cutoff, Perioperative outcomes

## Abstract

**Background:**

We aimed to determine a data-based annual hospital volume threshold for transurethral resection of the prostate (TURP), simple prostatectomy, holmium laser enucleation of the prostate (HoLEP), and thulium laser enucleation of the prostate (ThuLEP) and evaluate its clinical significance regarding perioperative morbidity.

**Materials and methods:**

We utilized the German Nationwide Inpatient Data (GRAND), provided by the Research Data Center of the Federal Statistical Office (2005–2022). Based on ROC analyses, the optimal annual hospital volume threshold to reduce perioperative incontinence, intensive care unit (ICU) admission, sepsis, transfusion, and hospital stay was identified for these surgeries.

**Results:**

A total of 1,084,650 TURP cases, 90,735 simple prostatectomy cases, 64,325 HoLEP cases, and 15,241 ThuLEP cases were included. For TURP, the annual hospital volume threshold to reduce perioperative morbidity was 266 cases for incontinence, 196 for ICU admission, 279 for sepsis, 241 for transfusions, and 139 for hospital stay. For simple prostatectomy, the annual hospital volume threshold to reduce perioperative morbidity was 22 cases for incontinence, 11 for ICU admission, 26 for sepsis, 23 for transfusions, and 32 for hospital stay. For HoLEP, the annual hospital volume threshold to reduce perioperative morbidity was 290 cases for incontinence, 120 for ICU admission, 140 for sepsis, 132 for transfusions, and 180 for hospital stay. For ThuLEP, the annual hospital volume threshold to reduce perioperative morbidity was 55 cases for incontinence, 56 for ICU admission, 217 for sepsis, 331 for transfusions, and 68 for hospital stay.

**Conclusion:**

The annual hospital volume threshold for improving perioperative outcomes in BPH surgery is high. Thus, centralization of benign prostatic hyperplasia surgery may be mandatory in some cases.

**Supplementary Information:**

The online version contains supplementary material available at 10.1007/s00345-026-06559-y.

## Introduction

Benign prostatic hyperplasia (BPH) is highly prevalent among men, with its occurrence rising steadily with age [1]. Approximately 20% of men with BPH will eventually undergo surgical intervention. Traditionally, transurethral resection of the prostate (TURP) has been the gold standard surgical therapy for BPH, since it is associated with a low risk of complications and high satisfaction rates [2]. Simple prostatectomy is also considered a standard surgical procedure [3]. Nevertheless, the landscape of available surgical treatment modalities has expanded significantly in recent years [4]. Holmium laser enucleation of the prostate (HoLEP) and thulium laser enucleation of the prostate (ThuLEP) are also considered among the first-line surgical treatment modalities for BPH [5].

Available epidemiological studies suggest the predominance of TURP for the management of small prostates and simple prostatectomy for larger prostates since both surgical procedures have a relatively short learning curve [6]. On the contrary, enucleation of the prostate is considered a demanding procedure with a steeper learning curve [7]. Nevertheless, accumulating evidence indicates that perioperative complications are more common after TURP and simple prostatectomy. Among them, approximately 5% are severe, prolonging hospital stay. The most important perioperative complications include incontinence, admission to the intensive care unit (ICU), sepsis, and transfusion [8].

Increased annual hospital volume has been associated with improved perioperative outcomes for major urological cancer surgeries such as radical cystectomy, nephrectomy, and prostatectomy [9]. High-volume centers are more likely to provide the required infrastructure and trained medical and paramedical personnel [10]. Additionally, experienced surgical teams can shorten the operative time, minimize postoperative complications, and manage intraoperative complications effectively [11]. Therefore, there might be a trend toward centralization of invasive BPH surgeries such as laser enucleation of the prostate [12]. Yet, no studies have attempted to identify an annual hospital caseload threshold for these surgeries that may reduce perioperative morbidity. Building on the previous notion, we aimed to determine a data-based annual hospital volume threshold after which perioperative morbidity improves for TURP, simple prostatectomy, HoLEP, and ThuLEP.

## Methods

### GeRmAn nationwide inpatient data (GRAND)

This study utilized data from the German Nationwide Inpatient Data (GRAND) registry obtained from the Federal Statistical Office of Germany. The GRAND registry includes data on all hospitalizations in Germany, excluding psychiatric, military, and forensic cases. We analyzed inpatient outcomes across Germany between 2005 and 2022. Since 2004, German hospitals have been required to systematically document patient information, including previous diagnoses, perioperative outcomes, surgical procedures, and other medical interventions. Diagnoses and perioperative outcomes are coded according to the International Statistical Classification of Diseases and Related Health Problems, 10th Revision, German Modification (ICD-10-GM), while surgical procedures and interventions are recorded using the German Procedure Classification (OPS).

These administrative data are mandatory for hospital reimbursement and are therefore submitted to the Institute for the Hospital Remuneration System. Researchers can access these data through the Research Data Center of the Federal Statistical Office upon formal request (agreement number: LMU-4710-2022).

### Selection criteria and statistical analysis

Patients who underwent TURP (OPS code: 5-601.1, 5-601.0), simple prostatectomy, open or minimally invasive (OPS code: 5-603), HoLEP (OPS code: 5-601.70), and ThuLEP (OPS code: 5-601.72) were included in the study. Additional procedures, comorbidities, and postoperative complications were identified using the corresponding ICD-10-GM and OPS codes. The primary objective of the study was to determine an annual hospital caseload threshold associated with improved inpatient outcomes. To identify this optimal threshold, receiver operating characteristic (ROC) analyses were performed, and the corresponding Youden’s index was calculated for each point along the ROC curve. The value that maximized Youden’s index was selected as the optimal cutoff for annual hospital caseload for perioperative, in-hospital morbidity outcomes. Morbidity outcomes included incontinence (transient early postoperative incontinence during hospital stay), ICU admission, length of hospital stay, transfusion, and sepsis. All categorical variables were reported as frequencies with proportions, while all continuous variables were reported as medians with interquartile range (IQR).

For each identified threshold, sensitivity, specificity, positive predictive value, and negative predictive value were also calculated. All statistical analyses were performed within the Research Data Center of the Federal Statistical Office using R scripts provided by our research team (source: RDC of the Federal Statistical Office and the Statistical Offices of the federal states, DRG Statistics 2005–2022, own calculations).

## Results

### Baseline characteristics

Between 2005 and 2022, a total of 1,084,650 TURP, 90,735 simple prostatectomy, 64,325 HoLEP, and 15,241 ThuLEP surgeries were performed. During hospital stay after TURP, a total of 39,510 (3.6%) incontinence cases, 16,599 (1.5%) ICU admissions, 8,488 (0.8%) sepsis cases, and 49,563 (4.6%) transfusions were recorded. The median length of hospital stay was 6 days (IQR: 4–8). During hospital stay after simple prostatectomy, a total of 3,860 (4.3%) incontinence cases, 7,246 (8%) ICU admissions, 804 (0.9%) sepsis cases, and 13,177 (15%) transfusions were recorded. The median length of hospital stay was 11 days (IQR: 9–14). During hospital stay after HoLEP, a total of 32,909 (4.5%) incontinence cases, 603 (0.9%) ICU admissions, 307 (0.5%) sepsis cases, and 1,175 (1.8%) transfusions were recorded. The median length of hospital stay was 4 days (IQR: 3–5). During hospital stay after ThuLEP, a total of 389 (2.6%) incontinence cases, 117 (0.8%) ICU admissions, 91 (0.6%) sepsis cases, and 362 (2.4%) transfusions were recorded. The median length of hospital stay was 3 days (IQR: 3–5). All perioperative outcomes are available in Table 1.

**Table 1:** Important perioperative outcomes of TURP, simple prostatectomy, HoLEP, and ThuLEP

Complication	TURP	Simple prostatectomy	HoLEP	ThuLEP
Incontinence	39,510 (3.6%)	3,860 (4.3%)	2,909 (4.5%)	389 (2.6%)
ICU admission	16,599 (1.5%)	7,246 (8%)	603 (0.9%)	117 (0.8%)
Sepsis	8,488 (0.8%)	804 (0.9%)	307 (0.5%)	91 (0.6%)
Transfusion	49,563 (4.6%)	13,177 (15%)	1,175 (1.8%)	362 (2.4%)
Length of hospital stay (days)	6 (4–8)	11 (9–14)	4 (3–5)	3 (3–5)

Variables are presented as median (interquartile range) or frequencies with proportions

*HoLEP* holmium laser enucleation of the prostate,* ICU* intensive care unit,* ThuLEP* thulium laser enucleation of the prostate,* TURP* transurethral resection of the prostate

### Annual hospital caseload threshold

All annual hospital caseload thresholds are presented in Table 2. For TURP, the ROC analyses suggested that the annual hospital volume threshold to reduce perioperative morbidity was 266 cases for incontinence, 196 for ICU admission, 279 for sepsis, 241 for transfusions, and 139 for hospital stay. The corresponding sensitivity, specificity, positive predictive value, and negative predictive value of these thresholds based on the ROC analyses are depicted in Data Supplement 1. For simple prostatectomy, the ROC analyses suggested that the annual hospital volume threshold to reduce perioperative morbidity was 22 cases for incontinence, 11 for ICU admission, 26 for sepsis, 23 for transfusions, and 32 for hospital stay. The corresponding sensitivity, specificity, positive predictive value, and negative predictive value of these thresholds based on the ROC analyses are depicted in Data Supplement 2.

**Table 2:** Annual hospital caseload threshold for important perioperative complications after TURP, simple prostatectomy, HoLEP, and ThuLEP, based on the maximum Youden’s index of the ROC analysis

Complication	TURP	Simple prostatectomy	HoLEP	ThuLEP
Incontinence	266	22	290	55
ICU admission	196	11	120	56
Sepsis	279	26	140	217
Transfusion	241	23	132	331
Length of hospital stay	139	32	180	68

*HoLEP* holmium laser enucleation of the prostate,* ICU* intensive care unit,* ThuLEP* thulium laser enucleation of the prostate,* TURP* transurethral resection of the prostate

For HoLEP, the ROC analyses suggested that the annual hospital volume threshold to reduce perioperative morbidity was 290 cases for incontinence, 120 for ICU admission, 140 for sepsis, 132 for transfusions, and 180 for hospital stay. The corresponding sensitivity, specificity, positive predictive value, and negative predictive value of these thresholds based on the ROC analyses are depicted in Data Supplement 3. For ThuLEP, the ROC analyses suggested that the annual hospital volume threshold to reduce perioperative morbidity was 55 cases for incontinence, 56 for ICU admission, 217 for sepsis, 331 for transfusions, and 68 for hospital stay. The corresponding sensitivity, specificity, positive predictive value, and negative predictive value of these thresholds based on the ROC analyses are depicted in Data Supplement 4.

## Discussion

The present nationwide analysis from Germany provides comprehensive data on the optimal annual hospital caseload of TURP, simple prostatectomy, HoLEP, and ThuLEP. Our findings indicate that an annual hospital caseload threshold of around 200 TURP cases, 20 simple prostatectomy cases, 150 HoLEP cases, and 100 ThuLEP cases was associated with lower perioperative morbidity and shorter hospital stay in ROC-based analyses. However, these associations should not be interpreted as evidence of a causal relationship between hospital volume and outcomes. Based on large-scale data, we demonstrate that increasing the volume of BPH invasive surgeries could optimize inpatient outcomes. Nevertheless, it seems that although the annual hospital volume threshold for improving perioperative outcomes in BPH surgery is high, the overall incidence of perioperative complications remains low.

Laser enucleation is a demanding procedure for beginners and has a steep learning curve [13]. Prolonged operating time and problems during enucleation increase the perioperative complications and seem to be the most important problems for beginners [14]. Accumulating evidence suggests that each surgeon should perform at least 50, and preferably over 100, procedures to master the technique [15]. This caseload may be even higher for hospitals; yet, no studies have assessed the overall hospital caseload for laser enucleation to improve perioperative outcomes. Interestingly, our findings indicate that HoLEP is associated with a higher annual hospital caseload threshold compared to ThuLEP. The latter might be explained by the fact that, in many cases, hospitals opting for ThuLEP have previously mastered HoLEP [16]. Therefore, fewer annual cases may be needed to improve perioperative outcomes after ThuLEP compared to HoLEP.

On the contrary, our analyses suggest that hospitals performing simple prostatectomy, open or minimally invasive, need only 20 annual cases to provide improved perioperative outcomes. The latter might explain why many hospitals opt for simple prostatectomy instead of laser enucleation for larger prostates [17]. Importantly, our findings indicate that a small annual hospital caseload is needed not only for open but also for minimally invasive simple prostatectomy. Still, given that prior epidemiological studies suggest higher rates of perioperative complications after open compared to minimally invasive simple prostatectomy, an increasing number of German hospitals are preferring robotic simple prostatectomy over HoLEP or ThuLEP [3, 8]. The latter might be explained by the small learning curve of robotic simple prostatectomy for hospitals performing robotic radical prostatectomy or open simple prostatectomy [18]. Still, it should be stressed that laser enucleation of the prostate is associated with better perioperative outcomes compared to minimally invasive simple prostatectomy, even for larger prostates [19].

Even though TURP remains the reference standard for all BPH surgical treatments [20], the current study indicates an annual hospital caseload threshold of around 200 TURP cases. This is significantly higher than simple prostatectomy and laser enucleation. The high threshold might reflect that TURP is widely performed across hospitals with varying levels of experience and case volumes. Consequently, a higher annual caseload may be required to achieve improved perioperative outcomes [21]. Importantly, this may explain why more surgeons and hospitals are choosing emerging minimally invasive surgical treatments for BPH [22]. Furthermore, the finding that approximately 200 TURP cases are required to improve perioperative outcomes may reflect the fact that more advanced procedures, such as simple prostatectomy or laser enucleation, are performed after mastering TURP [20]. Therefore, the higher TURP caseload threshold may primarily reflect the broader distribution of TURP across hospitals and surgeons with varying levels of expertise rather than the intrinsic procedural learning curve itself. Consequently, the identified thresholds should not be interpreted as definitive clinical standards but rather as data-driven estimates that require validation in future studies.

It should be highlighted that postoperative complications may be attributed to differences in surgeon expertise and case selection rather than true hospital caseload effects [21]. For example, junior trainees may perform a substantial proportion of TURP procedures, whereas laser enucleation or simple prostatectomy are often reserved for more experienced surgeons [22]. The absence of surgeon-level data, particularly regarding surgeon experience and annual surgeon caseloads, remains a major confounder when attempting to establish procedure-specific hospital volume thresholds associated with improved outcomes [23]. Yet, studies from major cancer surgeries highlight that increased annual hospital volume, and not surgeon volume, has been associated with improved perioperative outcomes [24, 25].

Although we provide, to our knowledge, the first study exploring the annual hospital caseload on perioperative, morbidity, and length of hospital stay in patients undergoing TURP, simple prostatectomy, HoLEP, and ThuLEP, it should be recognized that our findings were tempered by several limitations. Importantly, our results are based on administrative data and, thus, are prone to coding misclassification or inconsistencies. Of note, we restricted our morbidity analyses only to incontinence, sepsis, ICU admission, and transfusion. Similarly, the Clavien-Dindo classification could not be applied. Even though the billing data of the German nationwide inpatient database present a high degree of accuracy, several important cofounders, such as prior treatments, baseline functional parameters, operative indications, prostate volume, concomitant prostate cancer, operative time, patient laboratory findings (including PSA levels), as well as detailed patient comorbidity profiles (including ASA score or Charlson Comorbidity Index) and demographics, are not provided.

Accordingly, no data on the annual surgeon’s caseload could be retrieved. It should also be acknowledged that information on morbidity after hospital discharge, readmission rates, functional outcomes (including postvoid residual, Qmax, and IPSS), recurrence rates, as well as follow-up data, are not included in the GRAND study, limiting the extrapolation of our findings. Additionally, the use of ROC-derived Youden index thresholds to define annual hospital caseload thresholds may oversimplify what is likely a multifactorial relationship between hospital volume and perioperative outcomes. Given the very large sample size, statistically derived thresholds may not necessarily correspond to clinically meaningful differences in outcomes. Furthermore, the identified thresholds were not externally validated in an independent cohort and should therefore be interpreted as exploratory and hypothesis-generating. Hospital volume alone may not adequately capture procedural quality, especially without multivariable adjustment for important patient-, surgeon-, and procedure-related confounders. Lastly, as the findings are specific to the German healthcare system, caution is warranted in extrapolating these results to countries with different healthcare systems and BPH surgical practices.

The present high-volume, real-world data demonstrate that the annual hospital caseload to improve perioperative outcomes for TURP is around 200 cases, and for simple prostatectomy, only 20 cases. On the contrary, the annual hospital caseload for HoLEP is around 150 cases, and for ThuLEP, around 100 cases. Still, it should be highlighted that the total incidence of perioperative complications after invasive BPH surgeries is low. Overall, this analysis provides descriptive nationwide data on the association between annual hospital caseload and perioperative outcomes after BPH surgery. The identified thresholds may help generate hypotheses regarding volume–outcome relationships and facilitate the design of methodologically rigorous future studies. Whether these findings support centralization strategies for BPH requires further validation.

## Supplementary Information

Below is the link to the electronic supplementary material.

Supplementary Material 1

## Data Availability

No datasets were generated or analysed during the current study.
