# Supplementary material for: Hospital caseload thresholds for improved perioperative outcomes during transurethral resection or enucleation of the prostate: results from the GRAND study
Source: World J Urol. 2026 Jun 25;44(1):459. doi: 10.1007/s00345-026-06559-y (PMC13303316; doi:10.1007/s00345-026-06559-y)
Supplement: Supplementary file 1 — Supplementary Material 1 [file 345_2026_6559_MOESM1_ESM.docx]

Supplementary Material

Table of Contents

[Supplementary Material 1: Youden’s index to define the TURP annual hospital volume threshold that improves important perioperative outcomes 2](#_Toc231544070)

[Supplementary Material 2: Youden’s index to define the simple prostatectomy annual hospital volume threshold that improves important perioperative outcomes 3](#_Toc231544071)

[Supplementary Material 3: Youden’s index to define the HoLEP annual hospital volume threshold that improves important perioperative outcomes 4](#_Toc231544072)

[Supplementary Material 4: Youden’s index to define the ThuLEP annual hospital volume threshold that improves important perioperative outcomes 5](#_Toc231544073)

# Supplementary Material 1: Youden’s index to define the TURP annual hospital volume threshold that improves important perioperative outcomes

| **Complication** | **Hospital caseload threshold** | **Specificity** | **Sensitivity** | **NPV** | **PPV** |
| --- | --- | --- | --- | --- | --- |
| **Incontinence** | 266 | 0,18 | 0,84 | 0,97 | 0,04 |
| **ICU admission** | 196 | 0,38 | 0,7 | 0,99 | 0,02 |
| **Sepsis** | 279 | 0,16 | 0,87 | 0,99 | 0,01 |
| **Transfusion** | 241 | 0,24 | 0,79 | 0,96 | 0,05 |
| **Length of hospital stay** | 139 | 0,51 | 0,58 | 0,13 | 0,9 |

Supplementary Material 1: Annual hospital TURP caseload threshold for important perioperative complications, based on the maximum Youden's index of the ROC analysis. ICU: intensive care unit, TURP: transurethral resection of the prostate, NPV: negative predictive value, PPV: positive predictive value.

# Supplementary Material 2: Youden’s index to define the simple prostatectomy annual hospital volume threshold that improves important perioperative outcomes

| **Complication** | **Hospital caseload threshold** | **Specificity** | **Sensitivity** | **NPV** | **PPV** |
| --- | --- | --- | --- | --- | --- |
| **Incontinence** | 22 | 0,47 | 0,62 | 0,97 | 0,05 |
| **ICU admission** | 11 | 0,79 | 0,3 | 0,93 | 0,11 |
| **Sepsis** | 26 | 0,37 | 0,71 | 0,99 | 0,01 |
| **Transfusion** | 23 | 0,45 | 0,64 | 0,88 | 0,17 |
| **Length of hospital stay** | 32 | 0,97 | 0,37 | 0,47 | 0,95 |

Supplementary Material 2: Annual hospital simple prostatectomy caseload threshold for important perioperative complications, based on the maximum Youden's index of the ROC analysis. ICU: intensive care unit, NPV: negative predictive value, PPV: positive predictive value.

# Supplementary Material 3: Youden’s index to define the HoLEP annual hospital volume threshold that improves important perioperative outcomes

| **Complication** | **Hospital caseload threshold** | **Specificity** | **Sensitivity** | **NPV** | **PPV** |
| --- | --- | --- | --- | --- | --- |
| **Incontinence** | 290 | 0,7 | 0,6 | 0,97 | 0,09 |
| **ICU admission** | 120 | 0,63 | 0,55 | 0,99 | 0,01 |
| **Sepsis** | 140 | 0,57 | 0,59 | 1 | 0,01 |
| **Transfusion** | 132 | 0,6 | 0,54 | 0,99 | 0,02 |
| **Length of hospital stay** | 180 | 0,67 | 0,52 | 0,08 | 0,96 |

Supplementary Material 3: Annual hospital simple prostatectomy caseload threshold for important perioperative complications, based on the maximum Youden's index of the ROC analysis. HoLEP: holmium laser enucleation of the prostate, ICU: intensive care unit, NPV: negative predictive value, PPV: positive predictive value.

# Supplementary Material 4: Youden’s index to define the ThuLEP annual hospital volume threshold that improves important perioperative outcomes

| **Complication** | **Hospital caseload threshold** | **Specificity** | **Sensitivity** | **NPV** | **PPV** |
| --- | --- | --- | --- | --- | --- |
| **Incontinence** | 55 | 0,75 | 0,38 | 0,98 | 0,04 |
| **ICU admission** | 56 | 0,75 | 0,48 | 0,99 | 0,01 |
| **Sepsis** | 217 | 0,7 | 0,41 | 0,99 | 0,01 |
| **Transfusion** | 331 | 0,85 | 0,23 | 0,98 | 0,04 |
| **Length of hospital stay** | 68 | 0,36 | 0,84 | 0,1 | 0,97 |

Supplementary Material 4: Annual hospital simple prostatectomy caseload threshold for important perioperative complications, based on the maximum Youden's index of the ROC analysis. ICU: intensive care unit, NPV: negative predictive value, PPV: positive predictive value, ThuLEP: thulium laser enucleation of the prostate.
